# Deficits Associated With Loss of STIM1 in Purkinje Neurons Including Motor Coordination Can Be Rescued by Loss of Septin 7

**DOI:** 10.3389/fcell.2021.794807

**Published:** 2021-12-21

**Authors:** Sreeja Kumari Dhanya, Gaiti Hasan

**Affiliations:** ^1^ National Centre for Biological Sciences, Tata Institute of Fundamental Research, Bangalore, India; ^2^ SASTRA University, Thanjavur, India

**Keywords:** Ca2+ signaling, gene expression, climbing fibers, VGLUT2, SOCE, synaptic function, neurodegenerative disorders

## Abstract

Septins are cytoskeletal proteins that can assemble to form heteromeric filamentous complexes and regulate a range of membrane-associated cellular functions. SEPT7, a member of the septin family, functions as a negative regulator of the plasma membrane–localized store-operated Ca^2+^ entry (SOCE) channel, Orai in *Drosophila* neurons, and in human neural progenitor cells. Knockdown of STIM, a Ca^2+^ sensor in the endoplasmic reticulum (ER) and an integral component of SOCE, leads to flight deficits in *Drosophila* that can be rescued by partial loss of SEPT7 in neurons. Here, we tested the effect of reducing and removing SEPT7 in mouse Purkinje neurons (PNs) with the loss of STIM1. Mice with the complete knockout of STIM1 in PNs exhibit several age-dependent changes. These include altered gene expression in PNs, which correlates with increased synapses between climbing fiber (CF) axons and Purkinje neuron (PN) dendrites and a reduced ability to learn a motor coordination task. Removal of either one or two copies of the *SEPT7* gene in *STIM1* ^*KO*^ PNs restored the expression of a subset of genes, including several in the category of neuron projection development. Importantly, the rescue of gene expression in these animals is accompanied by normal CF-PN innervation and an improved ability to learn a motor coordination task in aging mice. Thus, the loss of SEPT7 in PNs further modulates cerebellar circuit function in *STIM1* ^*KO*^ animals. Our findings are relevant in the context of identifying SEPT7 as a putative therapeutic target for various neurodegenerative diseases caused by reduced intracellular Ca^2+^ signaling.

## Introduction

Septins (SEPT) constitute a family of filament-forming GTPases that can assemble into hetero-oligomeric complexes in cells and regulate various cellular functions such as cytokinesis (Kinoshita et al., 1997; Gladfelter et al., 2001; Kinoshita and Noda, 2001), cell polarity determination and maintenance (Drees et al., 2001; Faty et al., 2002; Irazoqui and Lew, 2004), microtubule and actin dynamics (Kinoshita et al., 1997; Finger, 2002; Surka et al., 2002; Nagata et al., 2003), membrane associations, cell movement (Finger et al., 2003), vesicle trafficking (Hsu et al., 1998), and exocytosis (Beites et al., 1999). There are thirteen septin-encoding genes in mice and humans that can be classified into four different subgroups based primarily on sequence homology. The subgroups are SEPT2 consisting of SEPT1, 2, 4, and 5; SEPT3 consisting of SEPT3, 9, and 12; SEPT6 consisting of SEPT6, 8, 10, 11, and 14; and SEPT7 which is encoded by a single gene (Kartmann & Roth, 2001; Macara et al., 2002; Kinoshita, 2003; Pan et al., 2014; Soroor et al., 2021). Functionally, septins from each subgroup occupy distinct positions in septin heteromers that can in turn assemble to form higher order septin filaments and structures (Sirajuddin et al., 2007; Mendonça et al., 2019; Soroor et al., 2021).

Regulation of cellular Ca^2+^ signaling by septins was first demonstrated in an siRNA screen in HeLa cells that identified mammalian septins of the SEPT2 class (SEPT2/4/5) as positive regulators of STIM/Orai-mediated store-operated Ca^2+^ entry (SOCE) (Sharma et al., 2013) and is further supported by recent studies (de Souza et al., 2021). Unlike the SEPT2 subclass, a mutant for the single SEPT7 gene in *Drosophila* was found to function as a negative regulator of SOCE. Heterozygotes of the *Drosophila SEPT7* mutant could rescue cellular and systemic phenotypes associated with neuronal knockdown of the SOCE molecules STIM/Orai in adult *Drosophila*. Subsequent genetic and cellular studies support the idea that SEPT7 function in human stem cell–derived neural precursors and differentiated neurons is similar to *Drosophila*. In both human neuronal cells and *Drosophila* neurons, SEPT7 orthologs prevent spontaneous Ca^2+^ entry through the SOCE channel, Orai (Deb et al., 2016; Deb et al., 2020).

Altered intracellular Ca^2+^ signaling has been associated with several neurodegenerative disorders in mouse models and in humans (Street et al., 1997; Bezprozvanny and Hayden, 2004; Van De Leemput et al., 2007; Klejman et al., 2009; Sun et al., 2014; Zhou et al., 2016; Klar et al., 2017; Ando et al., 2018; Czeredys, 2020). For example, mutations in a gene encoding the ER-Ca^2+^ release channel *IP*_*3*_ *R1* in humans are associated with spinocerebellar ataxias 15 and 29 (SCA 15 and 29), leading to cerebellar atrophy with the loss of Purkinje neurons and resulting in impaired coordination of movements (Hasan and Sharma, 2020). Loss of the gene encoding the ER-Ca^2+^ sensor STIM1 in mouse cerebellar Purkinje neurons affects their intrinsic excitability (Ryu et al., 2017), mGluR1-dependent synaptic transmission along with age-dependent changes that include a reduced ability to learn a motor coordination task (Hartmann et al*.*, 2014), and changes in synaptic connectivity and gene expression (Dhanya and Hasan, 2021). Interestingly, in a mouse model of familial Alzheimer’s disease, raising SOCE by overexpressing STIM2 rescued loss of mushroom spines in hippocampal neurons (Sun et al., 2014). These findings suggest that restoring intracellular Ca^2+^ homeostasis by modulating SOCE in the initial stages of neurodegenerative disease conditions could be a therapeutic strategy for the treatment of these neurodegenerative disorders. In this study, we investigated the cellular and behavioral effects of reducing SEPT7 and thus increasing spontaneous Ca^2+^ entry through SOCE channels, in mice with loss of STIM1 in Purkinje neurons.

## Materials and Methods

### Animals

All experimental procedures were conducted in accordance with the Institutional Animal Ethics Committee which is approved by the Control and Supervision of Experiments on Animals (CPCSEA), New Delhi, India. All transgenic mice used for the experiments were bred and maintained in the NCBS Animal Facility, Bangalore, India. The conditional Cre-lox system (Nagy, 2000; Kim et al., 2018) was used to generate a double knockout compound mouse strain with deletion of *STIM1* and *SEPT7* together in Purkinje neurons. Mice were obtained where *loxP* sites flanked the EF hand of the *STIM1* gene encoded by exon 2 (*STIM1 flox*; Oh-hora et al., 2008) and the GTP-binding P-loop of the *SEPT7* gene encoded by exon 4 (*SEPT7 flox;* Menon et al., 2014). *Pcp2 Cre* mice [B6.129-Tg (Pcp2-cre) 2Mpin/J-The Jacksons Laboratory, RRID: IMSR_JAX: 004146 (Barski et al., 2000)] that express Cre under the control of the PCP2 promoter, thus allowing Cre-mediated recombination exclusively in the Purkinje neurons, were used for the experiment. Triple transgenic mice, *STIM1* ^*flox/flox*^; *SEPT7* ^*flox/flox*^; *PCP*_*2*_^*Cre/+*^ (*STIM1* ^*PKO*^; *SEPT7* ^*PKO*^), were generated by crossing the homozygous double transgenic *STIM1* ^*flox/flox*^; *SEPT7* ^*flox/flox*^ with *STIM1* ^*flox/+*^; *PCP2-Cre* ^*cre/+*^ mice. Mice heterozygous for the *SEPT7* floxed and homozygous for the *STIM1* floxed allele with *PCP2 (L7)-Cre* are referred to as *STIM1* ^*PKO*^; *SEPT7* ^*PHet*^ (*STIM1* ^*flox/flox*^; *SEPT7* ^*flox/+*^; *PCP2-Cre* ^*cre/+*^). Double transgenic mice, *STIM1* ^*flox/flox*^; *SEPT7* ^*flox/+*^ and *STIM1* ^*flox/flox*^; *SEPT7* ^*flox/flox*^, were used as controls for heterozygous and homozygous *SEPT7* knockout strains, respectively. The offspring obtained were further genotyped by PCR of genomic DNA extracted from tail clippings. Primer pairs used to detect the wild-type *STIM1* gene (348 bp) and the floxed *STIM1* gene (399 bp) are from Oh-hora et al. (2008) and are given in Table 1. Primers used to detect the presence or absence of Cre are listed in Table 1, and the presence of the *Cre* transgene was determined by observing a product length of 421 bp (Hartmann et al., 2014). The wild-type *SEPT7* gene and the floxed *SEPT7* gene were confirmed using the primer pairs shown in Table 1. The product length for the wild-type *SEPT7* gene is 151 bp, whereas the product length for the floxed *SEPT7* gene is 197 bp (Menon et al., 2014).

**TABLE 1:** Primers used for genotyping transgenic mice and for quantitative real-time PCR.

Gene	Forward (5′>3′)	Reverse (5′>3′)
*Stim1* (DNA)	CGA​TGG​TCT​CAC​GGT​CTC​TAG​TTT​C	GGC​TCT​GCT​GAC​CTG​GAA​CTA​TAG​TG
*Cre* (DNA)	GCC​GAA​ATT​GCC​AGG​ATC​AG	AGC​CAC​CAG​CTT​GCA​TGA​TC
*SEPT7* (DNA)	CTT​TGC​ACA​TAT​GAC​TAA​GC	GGT​ATA​GGG​GAC​TTT​GGG​G
*Pcp2*	CCA​GGC​CAG​AAC​CCA​GAA​AG	CCC​AGG​TCG​TTT​CTG​CAT​TC
*Stim1*	ACA​ACT​GGA​CTG​TGG​ATG​AGG	TGG​TTA​CTG​CTA​GCC​TTG​GC
*Gabra6*	TGC​TGG​AAG​GCT​ATG​ACA​ACC	GTC​TGG​CGG​AAG​AAA​ACA​TCC
*Pvalb*	ATG​GGG​ACG​GCA​AGA​TTG​G	GCG​AGA​AGG​ACT​GAG​ATG​GG
*Calm1*	CGT​TCT​TCC​TTC​CTT​CGC​TCG	TTC​CTT​GGT​TGT​GAT​GGT​GCC
*Dlg4*	ACC​AAG​ATG​AAG​ACA​CGC​CC	TTC​CGT​TCA​CAT​ATC​CTG​GGG
*Robo2*	CTG​CCA​TCT​AGA​CCT​GAC​TCC	ACG​AGA​TCC​TTG​ACC​TTG​CC
*Map4*	AGC​CAG​GTT​GAA​GGT​ATC​CC	TGG​CTG​CTC​TGA​TAA​TCC​GG
*Gigyf2*	GGA​CCG​CAG​TGT​TAA​AAA​GAC​C	TCT​GCT​GCC​ATT​CTT​CTC​CG
*Atp1a3*	CTG​CCG​ACA​TGA​TTC​TGC​TGG	AGG​AAG​GGT​GTG​ATC​TCA​GGG
*Itpr1*	TGA​AGG​GGA​ACA​GAA​CGA​GC	AGG​CCG​ATT​CTT​TGT​TTC​TGC
*Orai3*	CCT​GTG​GCC​TGG​TTT​TTA​TC	GTG​CCC​GGT​GTT​AGA​GAA​TG
*Casq2*	AGC​CCA​ACG​TCA​TCC​CTA​AC	AGT​CGT​CTT​CTC​CTG​TAG​TCC
*S100b*	GAT​GTC​TTC​CAC​CAG​TAC​TCC​G	AGC​GTC​TCC​ATC​ACT​TTG​TCC
*Cacng5*	CTT​CCT​GTG​ATG​TGA​GGG​CG	CAA​AAG​TTG​GAG​TCG​AGC​GC
*Kctd17*	GGC​TCC​TCC​TAC​AAC​TAT​GGG	GGA​GGG​AGA​AAA​GGT​TAG​CGG
*Vamp1*	CCC​GTC​TCG​TTG​CAT​TCT​CC	GTC​ATG​TTG​GGA​GGA​GGA​CC
*Syt11*	CAA​GAG​GAA​CAT​TCA​GAA​GTG​C	CCT​GAG​AGA​CCG​GTG​ATA​TCC
*Setd6*	TGG​TTT​TGC​TGA​GCC​CTA​TCC	CCC​CTA​CCA​TCT​CCT​GTT​TGC
*Gapdh*	CTT​TGG​CAT​TGT​GGA​AGG​GC	TGC​AGG​GAT​GAT​GTT​CTG​GG

Sequences of primers used for standard PCR for genotyping transgenic mice and for quantitative real-time PCR for all sets of genes are listed in the table. Standard PCR is carried out on genomic DNA extracted from tail clippings of transgenic mice. The PCR product length of the wild-type *STIM1* gene and the floxed *STIM1* gene are 348 bp and 399 bp, respectively (Oh-hora et al., 2008). The presence of Cre is identified by a PCR product length of 421 bp (Hartmann et al., 2014). The wild-type *SEPT7* gene and the floxed *SEPT7* gene were confirmed by PCR product lengths of 151 bp and 197 bp, respectively (Menon et al., 2014). All primers used for qPCR were designed using primer 3 (http://bioinfo.ut.ee/primer3-0.4.0/). *Pcp2*, Purkinje cell protein 2; *Stim1*, stromal interaction molecule 1; *Gabra6*, gamma-aminobutyric acid type A receptor subunit alpha 6). *Pvalb*, parvalbumin; *Calm1*,calmodulin 1; *Dlg4*, discs large homolog 4; *Robo2*, roundabout guidance receptor 2; *Map4*, microtubule-associated protein 4; *Gigyf2*, GRB10-interacting GYF protein 2; *Atp1a3*, Na+/K+-ATPase transporting subunit alpha 3; *Itpr1*, inositol 1,4,5-trisphosphate receptor 1, *Orai3*—Orai3; *Casq2*, calsequestrin 2, *S100b*—S100B; *Cacng5*, calcium voltage-gated channel auxiliary subunit gamma 5; *Kctd17*, potassium channel tetramerization domain containing 17; *Vamp1*, vesicle-associated membrane protein 1; *Syt11*, synaptotagmin 11; *Setd6*, SET domain containing 6; *Gapdh*, glyceraldehyde 3-phosphate dehydrogenase.

### Microdissection and RNA Isolation

Sagittal cerebellar sections of about 250 µm thickness were obtained from mice aged 1 year using a vibratome (Leica, VT1200) and following isoflurane anesthesia and decapitation. The slices were sectioned in ice-cold cutting solution that contains the following reagents (in mM): 87 NaCl, 2.5 KCl, 0.5 CaCl_2_, 7 MgCl_2_, 1.25 NaH_2_PO_4_, 26 NaHCO_3_, 75 sucrose, and 10 glucose, bubbled with 95% O_2_ and 5% CO_2_. Cerebellar slices were then microdissected into Purkinje neuronal and molecular layer (PNL + ML) that was separated from the granular neuronal layer (GNL) with white matter under an illuminated stereomicroscope (Ryu et al., 2017).

RNA was isolated from PNL with ML and GNL with white matter using TRIzol according to the manufacturer’s protocol. Microdissected tissue was homogenized in 500 μl TRIzol (Invitrogen Cat# 15596026) using a micropestle homogenizer, and the samples were vortexed prior to proceeding with the RNA isolation protocol. Isolated RNA was analyzed by a NanoDrop spectrophotometer (Thermo Scientific) to check for its purity, followed by running it on a 1% Tris–EDTA agarose gel to check its integrity. For cDNA synthesis, approximately 500 ng of total RNA isolated was used per sample. DNase treatment was carried out with a reaction volume of 22.1 µl containing 500 ng of isolated RNA, 1 mM DTT, 0.5 U of DNase I (amplification grade), and 20 U of RNase inhibitor (RNase OUT) which was incubated at 37°C for 30 min, followed by heat inactivation at 70°C for 10 min. DNase-treated samples were further subjected to cDNA synthesis with 200 U of Moloney murine leukemia virus reverse transcriptase, 1 mM deoxyribonucleotide triphosphate, and 50 µM random hexamers in a final volume of 20 µl. The reaction mixture was then incubated at 25°C for 10 min, followed by treatment at 42°C for 60 min, and finally heat inactivation at 70°C for 10 min. All reagents used for this experiment were purchased from Invitrogen (Life Technologies).

### Real-Time Quantitative PCRs

Real-time quantitative PCRs (qPCRs) were performed with the KAPA SYBR FAST qPCR kit (Sigma-Aldrich Cat# KK4601) in a total volume of 10 μl on an ABI 7500 Fast machine (Applied Biosystems) which is operated with ABI 7500 software version 2. Primer 3 (http://bioinfo.ut.ee/primer3-0.4.0/) was used to design primers. Sequences of primers for all set of genes are listed in Table 1. The fold change of gene expression levels in experimental conditions relative to control was normalized according to the 2^−ΔΔCt^ method, where ΔΔCt = [(Ct (target gene)—Ct (GAPDH)] Expt.—[(Ct (target gene)—Ct (GAPDH)] control. GAPDH was used as a housekeeping gene.

### Immunohistochemistry

Mice were anesthetized and then transcardially perfused with 1X PBS, followed by perfusion with 4% PFA in 1X PBS. Brains were harvested following perfusion and postfixed with 4% PFA overnight, followed by cryoprotection in 30% sucrose in 1X PBS. The fixed cerebellum was then embedded in 5% low melting agar and sliced using a vibratome into 35-µm-thin sections. The cerebellar sections were washed in 1X PBS, blocked for 1 h at 4°C in 0.1% Triton X-100 and 5% normal goat serum, and stained with antibodies overnight at 4°C against guinea pig anti-VGLUT2 (1:1,000; Synaptic Systems Cat# 135 404, RRID: AB_887884). The sections were then washed in PBS-T (0.1% Triton X-100 in 1X PBS) thrice and incubated with secondary antibody goat anti-guinea pig which is coupled to Alexa Fluor 488 (Invitrogen Cat# A-11073, RRID: AB_2534117) for 1 h at room temperature. The slices were then washed in 1X PBS, mounted in Vectashield medium (Vector Labs, Cat#: H-1000), and imaged using an Olympus confocal microscope FV3000 with FV31S-SW 2.1 viewer software.

### Confocal Imaging and Image Analysis

Confocal images were captured using a confocal laser microscope (Olympus FV3000 laser scanning confocal microscope) with a 40X objective (PlanApo, NA 1.0; Olympus oil-immersion). Confocal images were acquired at 1.0-µm-thickness intervals with the frame size of 512 × 512 pixels. For estimation of VGLUT2 puncta along PC dendrites at proximal ends, Imaris software (Bitplane, v 9.1.2) was used (Kaneko et al., 2011). The Filament Tracer software (Auto Depth) of Imaris was used to trace each dendritic filament setting the largest diameter threshold at 3 µm and the smallest diameter at 1.86 µm. To quantify VGLUT2 puncta along Purkinje neuron dendrites, spot detection in Imaris software was used by setting the spot diameter threshold as 2 µm and the total distance close to the filament as 3 µm for proximal dendrites.

### Rotarod Test

The rotarod assay was performed by first habituating mice to a rotarod (IITC, model# 775, Series 8 Software) by providing a short training session where they were subjected to a constant speed of 5 rpm for 400 s. The mice were then tested for four trials a day for 5 days consecutively. Each session began from 5 rpm and finally attained 45 rpm, with a ramp speed at 240 s (Hartmann et al., 2014). The velocity of rotation was thus increased, keeping a constant acceleration of 9 rpm/min. The time at which each mouse fell off the rotarod was recorded for each session, and the mean latency on the rod was then calculated for all four trials for each mouse across 5 days of sessions.

### Statistical Analysis

Statistical analysis was performed using GraphPad Prism 7.0 or Origin 8.0 software. The statistical methods used in each experiment are mentioned in the figure legends. All bar graphs indicate means and standard error of means, and statistical significance was defined as *p* < 0.05 (*), highly significant for *p* < 0.01 (**) and *p* < 0.001 (***) as determined using paired Student’s *t*-test or one-way analysis of variance (ANOVA). In case of the rotarod test, two-way ANOVA, followed by Tukey’s multiple comparison test, was adopted for comparisons between groups.

## Results

### Generation of *STIM1-SEPT7* Double Knockout Mice

**FIGURE 1:**
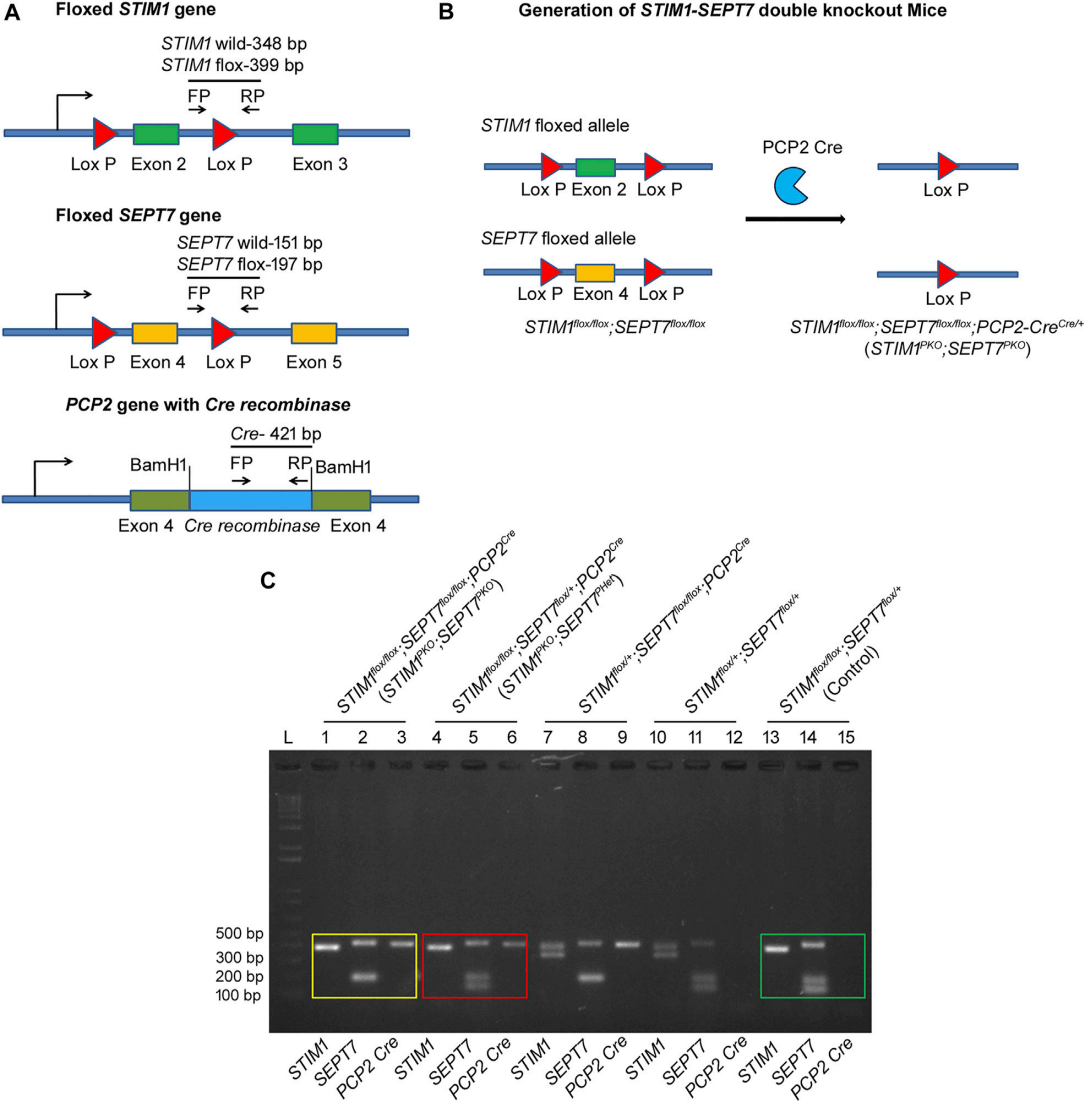
Generation of *STIM1*–*SEPT7* double knockout mice. **(A)** **(Top)** Schematic diagram with exon 2 of *STIM1* gene flanked by *loxP* recombination sites (red triangles). Cre-mediated recombination will result in the deletion of exon 2. **(Middle)** Schematic diagram with exon 4 of *SEPT7* gene flanked by *loxP* recombination sites (red triangles). **(Bottom)** Schematic diagram showing insertion of Cre-recombinase cDNA into the exon 4 of *PCP2* gene. Primers used for genotyping are indicated by arrows (FP, forward primer and RP, reverse primer). **(B)** Triple transgenic mouse strain *STIM1* ^*flox/flox*^; *SEPT7* ^*flox/flox*^; *PCP*_*2*_^*Cre/+*^ (*STIM1* ^*PKO*^; *SEPT7* ^*PKO*^) was generated by cross-breeding homozygous double transgenic *STIM1* ^*flox/flox*^; *SEPT7* ^*flox/flox*^ mice with *STIM1* ^*flox/+*^; *PCP2-Cre* ^*cre/+*^ mice to generate STIM1 and SEPT7 double knockout mice. **(C)** Agarose gel showing the genotyping of *STIM1–SEPT7* double knockout mice. PCRs of genomic DNA from *STIM1* ^*flox/flox*^; *SEPT7* ^*flox/flox*^; *PCP*_*2*_^*Cre/+*^ (*STIM1* ^*PKO*^; *SEPT7* ^*PKO*^) mice are shown on Lanes 1–3. PCRs were performed separately for each gene, and PCR products were loaded separately in different lanes. A single band at 399 bp indicates homozygous *STIM1 flox* (Lane 1), a band at 197 bp indicates homozygous *SEPT7 flox* (Lane 2), and the 421-bp band is from the *PCP2-Cre* allele (Lane 3). PCRs with genomic DNA from *STIM1* ^*flox/flox*^; *SEPT7* ^*flox/+*^; *PCP*_*2*_^*Cre/+*^ (*STIM1* ^*PKO*^; *SEPT7* ^*PHet*^) mice are shown in Lanes 4–6. Lanes 13–15 indicate genotyping of control mice *STIM1* ^*flox/flox*^ *; SEPT7* ^*flox/+*^. DNA band sizes are as described before. “L” denotes DNA ladder.

To understand if negative regulation of store-operated Ca^2+^ entry (SOCE) by SEPT7 is conserved among murine models and to investigate if motor deficits observed in *STIM1* knockout (*STIM1* ^*PKO*^) mice (Hartmann et al., 2014; Dhanya and Hasan, 2020) could be rescued by altering SEPT7 levels, we generated Purkinje neuron (PN)–specific compound *STIM1-SEPT7* double knockout mice strains using the conditional Cre-lox system (Figures 1A,B and methods). Exon 4, which encodes the GTP-binding P-loop of *SEPT7*, and exon 2, which encodes the EF hand of *STIM1*, were deleted in the progeny that included a *PCP2 (L7)-Cre* transgene as judged by the size of DNA fragments from appropriate PCRs from genomic DNA (Figure 1C and methods). Mice homozygous for the *SEPT7* floxed and *STIM1* floxed allele along with *PCP2 (L7)-Cre* are referred to as *STIM1* ^*PKO*^; *SEPT7* ^*PKO*^ [*STIM1* ^*flox/flox*^; *SEPT7* ^*flox/flox*^ *; PCP2* (*L7*)*-Cre* ^*cre/+*^, Figures 1B,C]. Mice heterozygous for the *SEPT7* floxed and homozygous for the *STIM1* floxed allele with *PCP2 (L7)-Cre* are referred to as *STIM1* ^*PKO*^; *SEPT7* ^*PHet*^ (*STIM1* ^*flox/flox*^; *SEPT7* ^*flox/+*^; *PCP2-Cre* ^*cre/+*^, Figure 1C). *STIM1* ^*flox/flox*^; *SEPT7* ^*flox/flox*^ and *STIM1* ^*flox/flox*^; *SEPT7* ^*flox/+*^ mouse strains in the absence of *PCP2* (*L7*)*-Cre* ^*cre/+*^ were used as controls for homozygous and heterozygous *SEPT7* knockout strains, respectively.

### Characterization of *SEPT7* and *STIM1* Expression Levels in *STIM1-SEPT7* Double Knockout Mice

**FIGURE 2:**
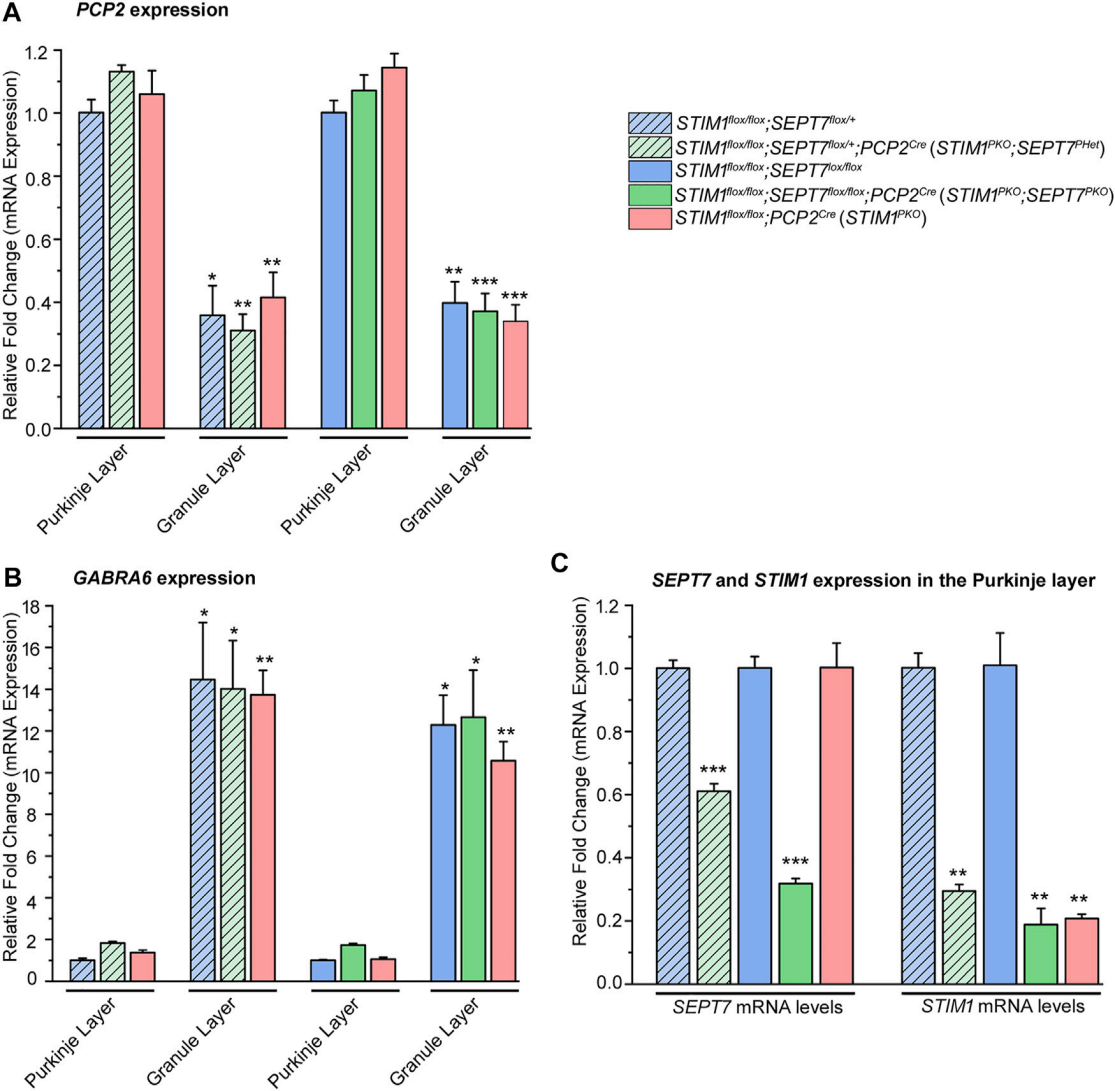
Characterization of *SEPT7* and *STIM1* expression levels. **(A)** Bar graphs indicating relative expression of Purkinje neuron marker, *PCP2* (Purkinje cell protein 2) normalized to *GAPDH* (glyceraldehyde 3-phosphate dehydrogenase) in samples of the Purkinje neuronal layer (PNL) and the granule layer (GL). *PCP2* expression levels of GL are relative to PNL for each genotype, and statistical significance is indicated above the bar graph. **(B)** Bar graphs with relative expression of granule layer marker, *GABRA6* (gamma-aminobutyric acid type A receptor subunit alpha 6) normalized to *GAPDH* levels in samples of the Purkinje neuronal layer and the granule layer. *GABRA6* expression levels of GL are compared relative to those of PNL for each genotype, and statistical significance is indicated above the bar graph. **(C)** Bar graphs indicating fold changes in expression of *SEPT7* and *STIM1* normalized to *GAPDH* from microdissected Purkinje neuronal layer of each genotype. Fold changes are relative to that of its respective controls, and statistical significance is indicated above the bar graph. Data are presented as mean ± SEM, *p < 0.05, **p < 0.01, ***p < 0.001; one-way ANOVA with post hoc Tukey’s test (*n* = 3 mice for all groups and *n* = 4 for *STIM1* ^*PKO*^; *SEPT7* ^*PHet*^, age—1-year-old mice).

Purkinje neurons present in the molecular layer were separated from the granule cell layer by hand microdissection of isolated cerebella from 1-year-old *STIM1-SEPT7* double knockout mice (Figure 2, green bars) and control mice of the appropriate genotypes (Figure 2, blue and pink bars). Enrichment of PNs and their separation from the granule cell layer was estimated by measuring relative gene expression levels of a Purkinje neuronal marker, Purkinje cell protein-2 (PCP2), and a granule cell layer marker GABA(A) receptor α6 subunit (GABRA6) (Boyden et al., 2006). Microdissected Purkinje layer samples from control and *STIM1-SEPT7* double knockout cerebella showed approximately three- to four-fold enrichment of PCP2 compared to the granule cell layer (Figure 2A) and a 10- to 14-fold lower expression of the granule cell marker GABRA6 (Figure 2B), indicating the minimal presence of granule neurons in the microdissected PN samples. The extent of SEPT7 knockdown in heterozygous and homozygous *SEPT7* knockout mouse strains was examined next. A significant reduction in *SEPT7* mRNA levels was observed in Purkinje layers isolated from the *STIM1* ^*PKO*^ *; SEPT7* ^*PHet*^ and *STIM1* ^*PKO*^ *; SEPT7* ^*PKO*^ animals as compared to control mice (Figure 2C). The expression of *STIM1* was also tested and found to be significantly reduced as expected in the three genotypes with homozygous *STIM1* ^*PKO*^. Thus, both heterozygous and homozygous conditions of *SEPT7* knockout in *STIM1* knockout backgrounds led to a significant reduction in *SEPT7* mRNA expression in PNs.

### Altered Expression of *SEPT7* in *STIM1* Knockout Mice Modulates Gene Expression in Purkinje Neurons

**FIGURE 3:**
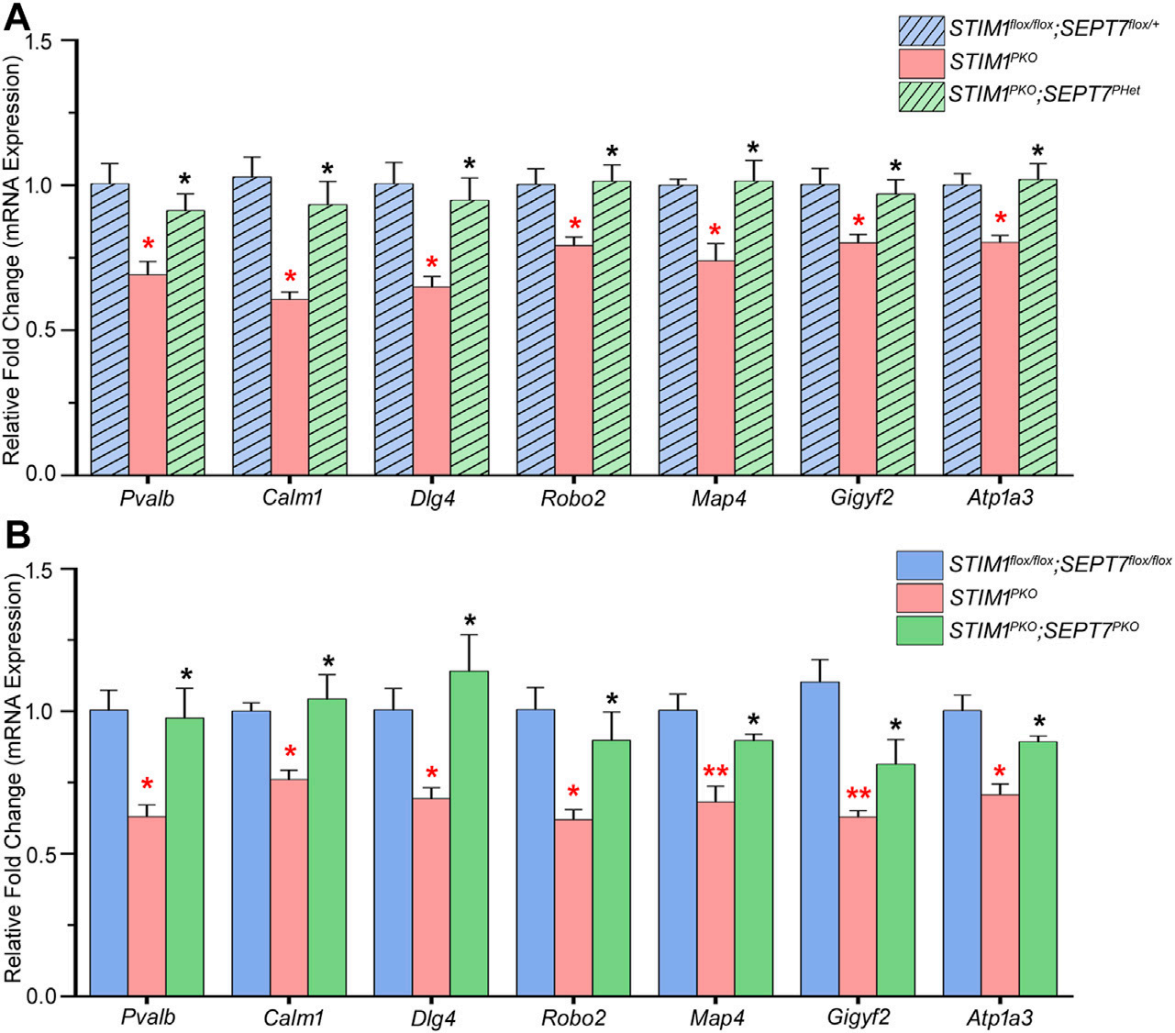
Partial or complete deletion of *SEPT7* can reverse the expression of a subset of genes that are downregulated by *STIM1* knockout in Purkinje neurons. **(A)** Bar graph with relative fold changes in expression of the indicated genes in the indicated genotypes. Red asterisks above each bar represent statistically significant change between *STIM1* ^*PKO*^ (pink bars) and the control genotype *STIM1* ^*flox/flox*^; *SEPT7* ^*flox/+*^ (light blue bars). Black asterisks above each bar represent statistically significant change between *STIM1* ^*PKO*^ (pink bars) and the experimental genotype, *STIM1* ^*PKO*^; *SEPT7* ^*PHet*^ (light green bars)*.* **(B)** Bar graph of relative gene expression changes in the indicated genotypes. Red asterisks over each bar represent statistically significant change between *STIM1* ^*PKO*^ (pink bars) and the control genotype *STIM1* ^*flox/flox*^; *SEPT7* ^*flox/flox*^ (blue bars)*.* Black asterisks above each bar represent a statistically significant change between *STIM1* ^*PKO*^ (pink bars) and *STIM1* ^*PKO*^; *SEPT7* ^*PKO*^ (green bars)*.* Fold changes were normalized to *GAPDH*. Data are presented as mean ± SEM, *p < 0.05, **p < 0.01; one-way ANOVA with post hoc Tukey’s test. All measurements are taken by qRT-PCR of cDNA prepared from RNA isolated from microdissected Purkinje layers (*n* = 3 mice for all groups and *n* = 4 for *STIM1* ^*PKO*^; *SEPT7* ^*PHet*^, age—1 year). *Pvalb*—Parvalbumin, *Calm1*—Calmodulin 1, *Dlg4*—Discs large homolog 4, *Robo2*—Roundabout guidance receptor 2, *Map4*—Microtubule-associated protein 4, *Gigyf2*—GRB10 interacting GYF Protein 2, *Atp1a3*—Na+/K+-transporting ATPase subunit alpha 3.

Age-dependent changes in gene expression have been reported recently in Purkinje neurons from *STIM1* ^*PKO*^ mice (Dhanya and Hasan, 2021). If reduced SEPT7 indeed raises Ca^2+^ entry in PNs, we hypothesized that gene expression changes that arise directly due to reduced SOCE should revert in PNs of *SEPT7* ^*PHet*^; *STIM1* ^*PKO*^ and *SEPT7* ^*PKO*^; *STIM1* ^*PKO*^ mice with age. This idea was tested by measuring the expression of a subset of genes, belonging to various gene ontology classes (Table 2), which are all significantly downregulated in PNs from 1-year-old *STIM1* ^*PKO*^ mice but not when tested in mice aged 4 months or less (Dhanya and Hasan, 2020). Expression levels of genes encoding the Ca^2+^-binding proteins parvalbumin (*Pvalb*) and calmodulin 1 (*Calm1*) were restored in PNs of both *STIM1* ^*PKO*^ *; SEPT7* ^*PHet*^ and *STIM1* ^*PKO*^ *; SEPT7* ^*PKO*^ mice when tested at 1 year (Figures 3A,B). Similarly, the expression of four genes, *Dlg4* (discs large homolog 4), *Robo2* (roundabout guidance receptor 2), *Map4* (microtubule-associated protein 4), and *Gigyf2* (GRB10 interacting GYF protein 2), was restored (Figures 3A,B). All four genes belong to the GO (gene ontology) category of neuron projection development (Table 2). In addition, reducing *SEPT7* in *STIM1* ^*PKO*^ PNs restored the expression of an ion channel gene *Atp1a3* encoding a Na^+^/K^+^-transporting ATPase subunit alpha 3 (Figures 3A,B). Thus, in 7 out of 16 genes tested (Figure 3 and Supplementary Figure S1), expression was restored significantly to control or near-control levels. The expression of genes like *Orai3* (SOCE channel), *Cacng5* (voltage-gated Ca^2+^ channel), *Vamp1* (vesicle-associated membrane protein 1), and *Syt11* (synaptotagmin 11) went up marginally in PNs from *STIM1* ^*PKO*^ *; SEPT7* ^*PKO*^ animals as compared to PNs from *STIM1* ^*PKO*^ mice (Supplementary Figure S1A). Although, due to technical limitations (Dhanya and Hasan 2021), we were unable to measure Ca^2+^ entry directly in PNs from *STIM1-SEPT7* double mutant conditions, these data support the idea that either reduction (*SEPT7* ^*PHet*^) or loss of SEPT7 (*SEPT7* ^*PKO*^) in PNs allows spontaneous extracellular Ca^2+^ entry, as observed previously in human stem cell–derived precursors and differentiated neurons (Deb et al., 2020). This putative mode of Ca^2+^ entry in PNs might partially compensate for loss of STIM1-mediated SOCE and concomitant gene expression changes.

**TABLE 2:** Table with downregulated genes identified under enriched GO biological and cellular process.

GO Term	Pathway	LogP	Genes
GO:0010975	Regulation of neuron projection development	−4.69	*Dlg4*, *Robo2*, *Gigyf2*, *S100b*, *Map4*, *Itpr1*, *Kctd17*
GO:0098984	Neuron to neuron synapse	-4.60	*Dlg4*, *Itpr1*, *Map4*, *Cacng5*, *Syt11*, *Atp1a3*, *Vamp1*
GO:0006898	Receptor-mediated endocytosis	−4.19	*Atp5b*, *Dlg4*, *Cacng5*, *Syt11*
GO:0090316	Positive regulation of intracellular protein transport	−4.10	*Syt11*, *Vamp1*, *Itpr1*
GO:0032469	Endoplasmic reticulum calcium ion homeostasis	−3.02	*Itpr1*, *Orai3*, *Dlg4*, *Casq2*, *S100b*, *Atp1a3*, *Cacng5*, *Kctd17*
GO:0061024	Membrane organization	−2.48	*Dlg4*, *Vamp1*, *Cacng5*, *Syt11*
GO:0098793	Presynapse	−2.35	*Calm1*, *Dlg4*, *Itpr1*, *Syt11*, *Atp1a3*

GO classification of biological and cellular processes of selected genes downregulated upon *STIM1* knockout in PNs. Enriched categories with associated GO term, log *p* value, and lists of genes associated with each process and tested here are shown. Metascape was used for gene enrichment using parameters specific for *Mus musculus*, with a *p* value cutoff as 0.01 (data from Dhanya and Hasan, 2021).

### Loss of SEPT7 Modulates Synaptic Connectivity Among Climbing Fiber Axons and Purkinje Neuron Dendrites in *STIM1* Knockout Purkinje Neurons

**FIGURE 4:**
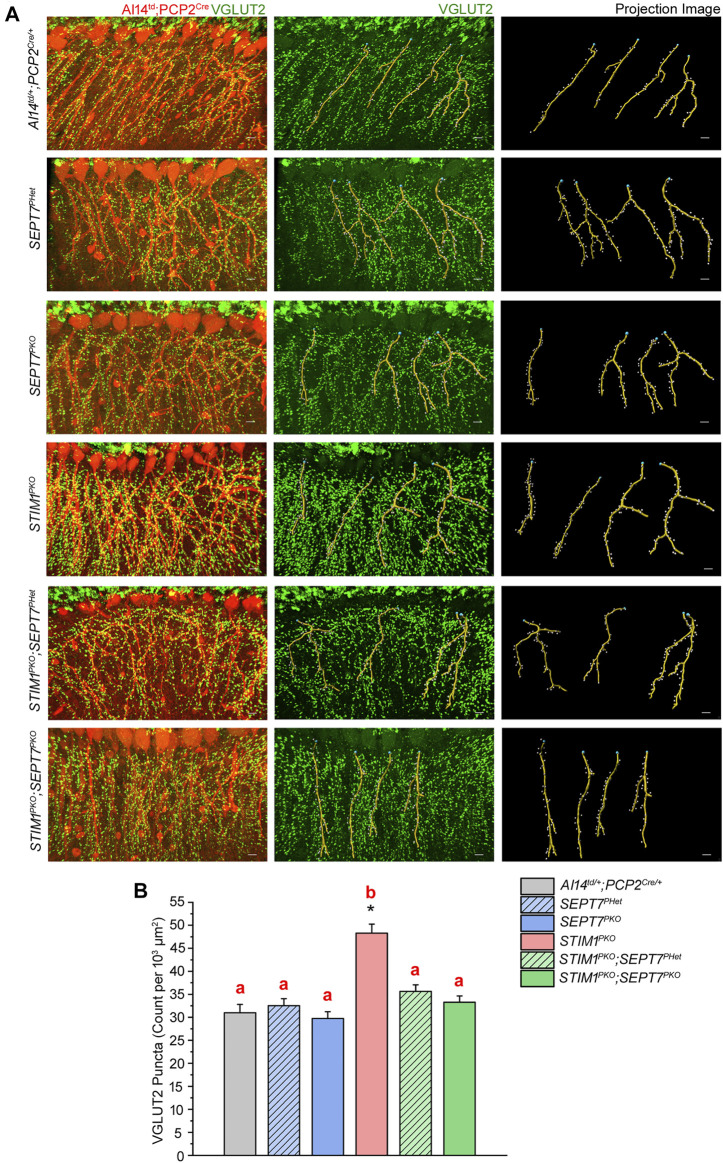
Excess innervation between climbing fibers and Purkinje neuron dendrites in *STIM1* mice can be reversed by reduction and loss of *SEPT7.* **(A)** Immunohistochemical images and quantitative analysis of climbing fiber innervations on the proximal dendrites of Purkinje neurons in the indicated mice genotypes. **(Left panel)** PN soma and proximal dendrites with Td tomato expression (red). VGLUT2 puncta are visible as green dots along the dendrites; **(middle panel)** VGLUT2 puncta (green) with projection images of dendritic filaments (yellow) obtained computationally using Imaris software and **(right panel)** projection images from Imaris analysis with dendritic filaments marked in yellow and VGLUT2 puncta as white dots. Scale bars are 10 μm. **(B)** Bar graph depicting the density of VGLUT2 puncta (count per 10^3^ μm^2^) presents at the proximal dendritic regions of PNs of the indicated mice genotypes. Quantification of VGLUT2 puncta density was from three mice of each genotype, all aged 1 year, and 27 or more PNs from each genotype. Data are presented as mean ± SEM; one-way ANOVA with post hoc Tukey’s test; *p < 0.00001. Same alphabets above the bar graphs represent statistically indistinguishable groups, and a different alphabet represents a statistically different group with the minimal significance of *p <* 0.05.

Next, we investigated if reducing or abolishing SEPT7 from *STIM1* ^*PKO*^ Purkinje neurons modulates synaptic connectivity between climbing fibers and Purkinje neurons. Climbing fibers (CFs) are axonal projections from inferior olive neurons. They innervate Purkinje neurons dendrites through glutamatergic synapses that express VGLUT2 (vesicular glutamate transporter type 2; Fremeau et al., 2001). Activity from CFs greatly influences individual Purkinje neurons and cerebellar output (Smeets and Verbeek, 2016). Excess CF-PN innervation has been seen in a number of genetic mouse models with impaired Ca^2+^ signaling and reduced motor coordination (Alba et al., 1994; Chen et al., 1995; Kano et al., 1995; Kashiwabuchi et al., 1995; Kano et al., 1997; Hashimoto et al., 2001). Moreover, *STIM1* ^*PKO*^ mice at 1 year exhibit increased CF-PN synapse numbers that correlate with reduced motor coordination (Dhanya and Hasan, 2020). To evaluate the density and distribution of CF-PN synapses, we quantified VGLUT2 (vesicular glutamate transporter type 2) puncta along the proximal dendrites of PNs (Figure 4). As described previously, a significant increase in VGLUT2 puncta is observed along the proximal dendrites of *STIM1* ^*PKO*^ PNs as compared with the control genotype of *AI14* ^*td*^; *PCP2* ^*Cre*^ (Figures 4A,B; Dhanya and Hasan, 2021). Importantly, the density of VGLUT2 puncta along the PN dendrites of both *STIM1* ^*PKO*^; *SEPT7* ^*PHet*^ and *STIM1* ^*PKO*^; *SEPT7* ^*PKO*^ mice was comparable to that of the control mice. There was no change in the density of VGLUT2 puncta on PN dendrites of either *SEPT7* ^*PHet*^ or *SEPT7* ^*PKO*^ mice (Figures 4A,B). Restoration of CF-PN innervation upon reducing SEPT7 in STIM1 knockout Purkinje neurons suggests that Ca^2+^ homeostasis impacts synaptic plasticity and together the two could alter cerebellar circuit function.

### Reduced SEPT7 Levels Improve Motor Performance in *STIM1* Knockout Mice

**FIGURE 5:**
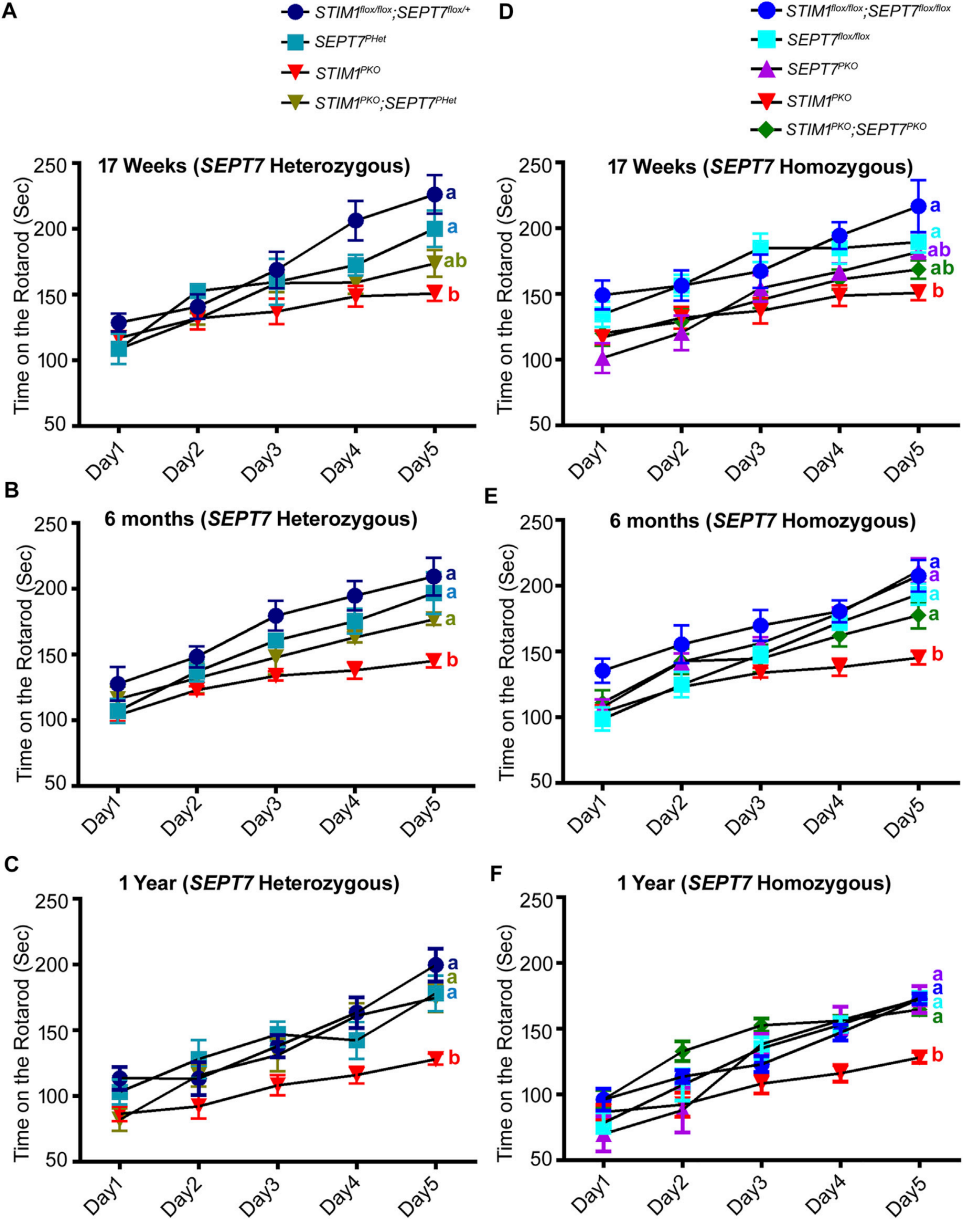
Reduction and loss of SEPT7 rescue loss of motor learning and coordination arising from loss of STIM1 in Purkinje neurons mean latency times on the rotarod are shown for the indicated genotypes at **(A, D)** 17 weeks, **(B, E)** 6 months, and **(C, F)** 1 year of age. The number of mice tested for each genotypes is *STIM1* ^*flox/flox*^; *SEPT7* ^*flox/+*^ (*n* = 6), *SEPT7* ^*PHet*^ (*n* = 4), *STIM1* ^*PKO*^ (*n* = 7), *STIM1* ^*PKO*^; *SEPT7* ^*PHet*^ (*n* = 5), *STIM1* ^*flox/flox*^; *SEPT7* ^*flox/flox*^ (*n* = 6), *SEPT7* ^*flox/flox*^ (*n* = 8), *SEPT7* ^*PKO*^ (*n* = 6), *STIM1* ^*PKO*^ (*n* = 7), *STIM1* ^*PKO*^ *; SEPT7* ^*PKO*^ (*n* = 8). Same alphabets at the end of line plots represent statistically indistinguishable groups, the color of the alphabets denotes the respective line plots, and different alphabets represent *p <* 0.05. Two-way ANOVA, a post hoc test, followed by Tukey’s multiple comparisons test were used.

A well-established readout of PN and cerebellar function is the ability to learn a motor coordination task such as time spent on a rotarod with increasing rotational speed (Koopmans et al., 2007; Shiotsuki et al., 2010; Stroobants et al., 2013). Loss of STIM1 in PNs reduces the ability to perform this motor learning task (Hartmann et al., 2014). Further impairment is seen with age both in controls and in *STIM1* ^*PKO*^ animals (Dhanya and Hasan, 2021). Restored expression of a subset of genes and rescue of CF-PN connectivity motivated us to compare the motor learning deficits of *STIM1* ^*PKO*^ mice with *STIM1* ^*PKO*^; *SEPT7* ^*PHet*^ and *STIM1* ^*PKO*^; *SEPT7* ^*PKO*^ mice, along with their appropriate genetic controls in the rotarod assay across various ages. Initially, we tested motor learning in mice with either partial or complete loss of SEPT7 in Purkinje neurons (Figure 5). Motor learning deficits were not observed in *SEPT7* ^*PHet*^ and *SEPT* ^*PKO*^ mice at 17 weeks, 6 months, or 1 year of age (Figures 5A–F), suggesting the limited function of SEPT7 in adult PNs. Next, we tested motor learning in the double mutant combinations of *STIM1* ^*PKO*^; *SEPT7* ^*PHet*^ and *STIM1* ^*PKO*^; *SEPT7* ^*PKO*^ mice. *STIM1* ^*flox/flox*^, *SEPT7* ^*flox/+*^, and *SEPT7* ^*PHet*^ were the controls for *STIM1* ^*PKO*^; *SEPT7* ^*PHet*^ mice, whereas *STIM1* ^*flox/flox*^, *SEPT7* ^*flox/flox*^, and *SEPT7* ^*PKO*^ were controls for *STIM1* ^*PKO*^; *SEPT7* ^*PKO*^ mice. The same batch of mice was tested at 17 weeks, 6 months, and 1 year of age. At all ages, tested motor learning in *STIM1* ^*flox/flox*^, *SEPT7* ^*flox/+*^, and *SEPT7* ^*PHet*^ mice is significantly better than in *STIM1* ^*PKO*^ mice (Figure 5A). For example, at 17 weeks, *STIM1* ^*flox/flox*^; *SEPT7* ^*flox/+*^ control mice improved their latency on the rotarod from 128.8 ± 6.9 s on the 1st day to 226.3 ± 14.7 s on the 5th day (Figure 5A), whereas *STIM1* ^*PKO*^ mice showed similar performance as that of control mice on day 1 (mean latency of 117.1 ± 5.1 s) but did not improve to the same extent (150.9 ± 5.6 s, Figures 5A,B) as the controls over 5 days of training. Performance of *STIM1* ^*PKO*^ *; SEPT7* ^*PHet*^ mice at 17 weeks is not statistically indistinguishable from either controls or *STIM1* ^*PKO*^ animals (Figure 5A). However, the age performance of *STIM1* ^*PKO*^; *SEPT7* ^*PHet*^ mice improves on the rotarod as evident at 6 months (Figure 5B; Supplementary Figure S2) and 1 year (Figure 5C). A similar age-dependent improvement in motor coordination learning is evident in *STIM1* ^*PKO*^ *; SEPT7* ^*PKO*^ homozygotes from 17 weeks to 1 year (Figures 5D–F; Supplementary Figure S2, Supplementary Video S1). However, as compared to *STIM1* ^*PKO*^; *SEPT7* ^*PHet*^ mice (Figure 5C), the *STIM1* ^*PKO*^; *SEPT7* ^*PKO*^ mice (Figure 5F) exhibit an overall reduction in motor learning with age, which is also evident for the control genotypes and is possibly due to the sustained loss of SEPT7 from PNs. Thus, deleting either single or both copies of *SEPT7* in PNs of *STIM1* ^*PKO*^ mice partially rescues their motor learning deficits.

## Discussion

**FIGURE 6:**
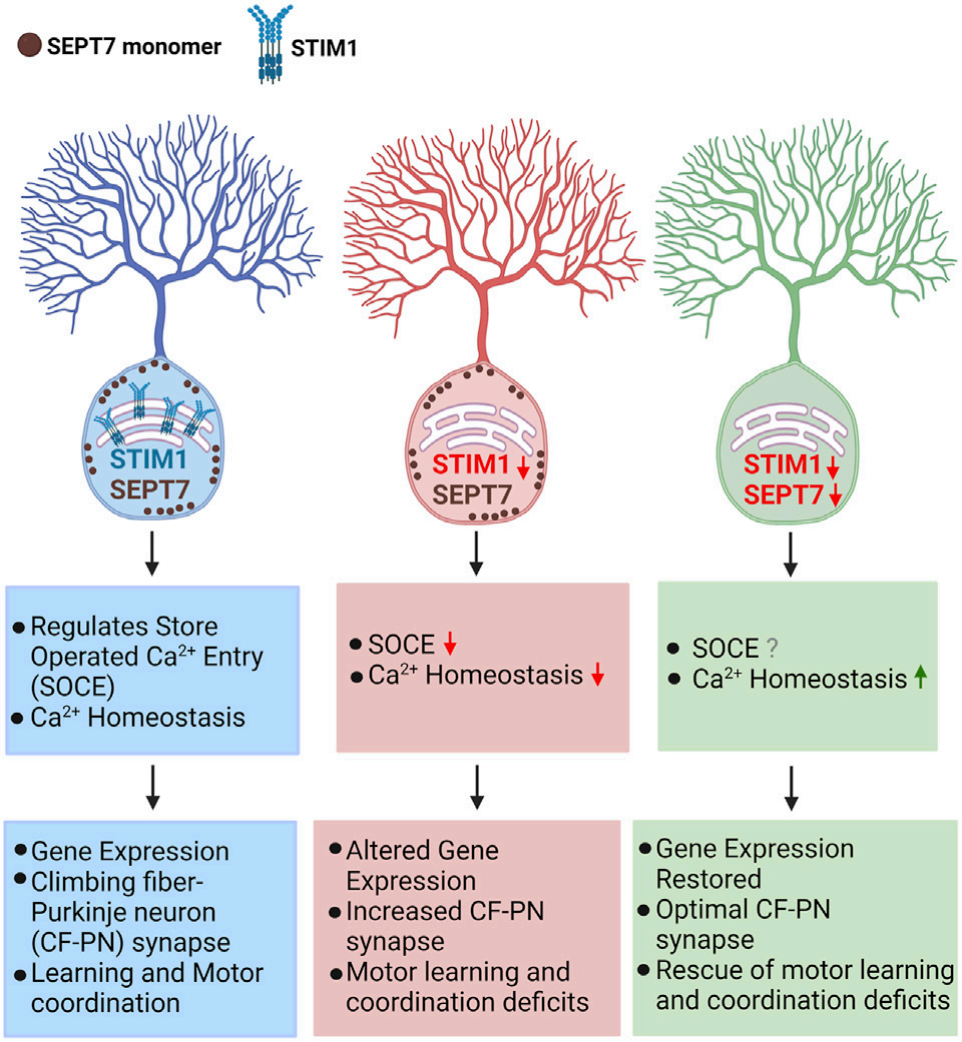
Cellular and behavioral effects of reducing SEPT7 in mice with loss of STIM1 in Purkinje neurons loss of STIM1 in Purkinje neurons attenuate Ca^2+^ entry and affect ER-Ca^2+^ refilling which in turn suppresses mGluR1-stimulated Ca^2+^ signals and Purkinje neuron excitability (Hartmann et al., 2014; Ryu et al., 2017; Dhanya and Hasan, 2021). These changes affect gene expression and optimal synaptic connectivity of cerebellar Purkinje neurons with age (Dhanya and Hasan, 2021). Reducing or abolishing *SEPT7* in *STIM1* ^*KO*^ PNs restored gene expression which is accompanied by normal CF-PN innervation and an improved ability to learn a motor coordination task in aging mice. [Model created using Biorender (BioRender.com).]

In this study, we show that partial or complete deletion of SEPT7 from adult murine Purkinje neurons reverses multiple phenotypes associated with PN-specific loss of the ER-Ca^2+^ sensor and SOCE protein STIM1. The phenotypes reverted include changes in gene expression in part, expression of VGLUT2 puncta that serve as a marker for altered neuronal connectivity between climbing fiber axons and PN dendrites, and the ability to learn a motor coordination task in aging mice. From a previous study, we know that loss of motor coordination is seen as early as 17 weeks of age in *STIM1* ^*PKO*^ mice, whereas changes in CF-PN synapse numbers and changes in gene expression become evident in mice aged 1 year (Dhanya and Hasan, 2021). *STIM1* ^*PKO*^ *–SEPT* ^*PHet/PKO*^ mice also exhibit early motor learning deficits. Importantly, the progression of these deficits undergoes reversal with age (Figure 5, Supplementary Figure S2, Supplementary Video S1). Taken together, these findings support the idea that reduction/loss of SEPT7 in adult PNs reverts key age-dependent changes in neuronal Ca^2+^ homeostasis that occurs from loss of STIM1. Consequently, there is reversion of other age-dependent cellular (VGLUT2 puncta) and molecular (gene expression) deficits, and this reversion correlates with better learning of motor coordination with age in *STIM1* ^*PKO*^ *–SEPT7* ^*PHet/PKO*^ mice (Figure 6). The reversion of motor coordination deficits observed in mice is in agreement with previous findings in *Drosophila* where partial genetic depletion of SEPT7 could rescue flight deficits due to STIM knockdown (Deb et al., 2016). Early changes (17 weeks) in mGluR1-dependent Ca^2+^ transients, of which STIM1 is an integral component, are very likely not restored by loss/reduction of SEPT7. The status of mGluR1-initiated Ca^2+^ signals in PNs from *STIM1* ^*PKO*^ *–SEPT7* ^*PHet/PKO*^ mice needs further investigation.

### SEPT7 May Function as a Monomer for Regulating Ca^2+^ Entry at the Plasma Membrane

Most cellular functions of septins are associated with their ability to form filaments and higher order cytoskeletal structures (Marquardt et al., 2019). Recent findings demonstrate that filament formation requires linear hexamers/octamers consisting of SEPT7 at the core, followed by other SEPT subunits in a specific order (Mendonça et al., 2019; Soroor et al., 2021). Differential regulation of SOCE by SEPT2/4 (positive regulators; Sharma et al., 2013; de Souza et al., 2021) and SEPT7 (negative regulator; Deb et al., 2016; Deb et al., 2020) is difficult to reconcile with SEPT7 as the core subunit of a minimally functional septin multimer. Unicellular organisms such as *Chlamydomonas reinhardtii* encode a single gene for septin (Pinto et al., 2017), raising the possibility that monomeric septin function may precede septin filament formation. For the negative regulation of SOCE channels, we propose that SEPT7 very likely functions as a monomer (Figure 6). At this stage, however, there is no direct evidence demonstrating the presence of SEPT7 monomers near the ER-PM junctions where SOCE is known to occur.

The mechanism by which SEPT7 regulates Ca^2+^ entry remains poorly understood. In human stem cell–derived neurons, spontaneous Ca^2+^ entry by loss of SEPT7 requires the polybasic N-terminal region known to interact with membrane-localized phospholipids such as PIP2 and PIP3 (Zhang et al., 1999; Deb et al., 2020). We predict that the interaction of SEPT7 monomers with membrane phospholipid(s) prevents the opening of a STIM1-regulated Ca^2+^ entry channel in PNs. Concomitant loss of SEPT7 with STIM1 possibly allows a low level of spontaneous Ca^2+^ entry through such channels and thus restores PN Ca^2+^ homeostasis and associated long-term deficits.

Among the deficits tested here, we did not find any significant change in *SEPT7* ^*PKO*^ animals, suggesting that in mature PNs, septin filaments have either no role or a very minor role. Possible effects of SEPT7 on dendritic branching were not investigated though a role for SEPT7 in regulating spine morphogenesis and dendrite development during neuronal maturation has been demonstrated in hippocampal neurons (Tada et al., 2007). SEPT7 monomers are possibly one among several regulators of STIM1-mediated Ca^2+^ entry, and presumably, their loss can be compensated by other regulators of Ca^2+^ signaling in PNs.

### Gene Expression Changes in Purkinje Neurons With Loss of STIM1, and Restored by SEPT7, Are Associated With Neurodegeneration

Purkinje neurons express a range of Ca^2+^-binding proteins, Ca^2+^ channels, Ca^2+^-dependent kinases, and phosphatases that not only tune PN excitability but also help maintain cellular Ca^2+^ homeostasis, regulate different Ca^2+^-dependent processes, and modulate multiple inputs received by PNs (Prestori et al., 2020). Changes in gene expression by loss of STIM1 suggest that the maintenance of Ca^2+^ homeostasis by STIM1 is required for appropriate age-dependent expression of multiple genes. Restored expression of genes encoding the Ca^2+^-binding proteins parvalbumin (*Pvalb*) and calmodulin 1 (*Calm1*) (Figure 3) is of interest because parvalbumin is expressed abundantly in PNs (Caillard et al*.*, 2000; Schwaller et al., 2002) and significant reduction in parvalbumin expression is reported from PNs of SCA1 (spinocerebellar ataxia 1) patients (Vig et al., 1996) and transgenic mice carrying the human SCA1-causing gene (Vig et al., 1998). Calmodulin 1, which is an EF hand containing Ca^2+^-binding protein, is known to regulate the activity of several Ca^2+^-regulated enzymes such as αCaMKII and βCaMKII that are required for cerebellar long-term depression (LTD) and motor learning (Hansel et al., 2006; Van Woerden et al., 2009). Reduced calmodulin levels are reported in the cerebellar vermis of the spontaneously ataxic mouse, *Pogo* (Lee et al., 2011).

The expression of certain genes classified under the GO category of neuron projection development is also restored in *STIM1* ^*PKO*^ *–SEPT* ^*PHet/PKO*^ mice (Figure 3). Dlg4 is a major scaffolding protein in the excitatory postsynaptic density that regulates synaptic strength (Kim et al*.*, 2004; Funke et al., 2005; Cheng et al*.*, 2006), and previous work has reported impaired motor coordination in *Dlg4* knockout mice (Feyder et al., 2010). Robo2 is a transmembrane receptor for the secreted molecule slit homolog 2 protein (Slit2) and plays an important role in axon guidance and cell migration (Ma and Tessier-Lavigne, 2007; Giovannone et al., 2012). It is highly expressed by PNs during dendritic arbor development, and PN-specific *Robo2*-deficient mice exhibit gait alterations (Gibson et al., 2014). Gigyf2 interacts with an adapter protein Grb10, which binds to activated IGF-I and insulin receptors (Giovannone et al., 2003; Holt and Siddle, 2005). Mice heterozygous for *Gigyf2* exhibit motor dysfunction (Giovannone et al., 2009). Loss of SEPT7 however does not restore the expression of every gene downregulated in *STIM* ^*PKO*^ PNs. This is possibly because reduced expression of some downregulated genes may be many steps downstream of Ca^2+^ entry, and slow restoration of PN Ca^2+^ homeostasis in either *SEPT7* ^*PHet*^; *STIM1* ^*PKO*^ or *SEPT7* ^*PKO*^;*STIM1* ^*PKO*^ animals may be insufficient to revert such changes. Moreover, SEPT7 may not restore precise Ca^2+^ dynamics related to STIM1 signaling that may be necessary for the expression of certain genes.

### Climbing Fiber–Purkinje Neuron Synaptic Connectivity and Motor Behavior

Climbing fibers provide one of the major excitatory inputs to Purkinje neurons (Lin et al., 2014). Climbing fiber–Purkinje neuron (CF-PN) synaptic wiring influences processing and integration of information at the PN dendrites essential for proper control of cerebellar motor learning and coordination (Ichikawa et al., 2016). The distribution of CF-PN synapses on Purkinje dendrites is regulated and required for the proper physiological function of Purkinje neurons (Watanabe, 2008). Abnormal CF-PN innervation has been reported in various genetic mouse models with impaired Ca^2+^ signaling and motor coordination deficits (Alba et al., 1994; Chen et al., 1995; Kano et al., 1995; Kashiwabuchi et al., 1995; Kano et al., 1997; Hashimoto et al., 2001). Defective CF–PN connections have also been observed in the initial stages of Purkinje neuron degeneration in various forms of spinocerebellar ataxias and in conditions of abnormal activity in climbing fibres (Cheng et al., 2013; Koeppen et al., 2013; Helmich et al., 2013). Restoration of CF–PN innervation in *STIM1* ^*PKO*^ *–SEPT7* ^*PHet/PKO*^ mice (Figure 4) is thus an important observation and very likely causative in the restoration of motor learning with age.

In conclusion, our data demonstrate that partial or complete loss of SEPT7 in *STIM1* knockout Purkinje neurons could restore age-dependent gene expression changes observed in *STIM1* ^*PKO*^ PNs. This is accompanied by normal climbing fiber–Purkinje neuron synaptic connectivity and an improved ability to learn and perform motor coordination task in aging mice. These findings suggest that loss of SEPT7 in Purkinje neurons allows spontaneous extracellular Ca^2+^ entry, as reported previously in *Drosophila* neurons (Deb et al., 2016) and in human neural progenitor cells (Deb et al., 2020), and this mode of Ca^2+^ entry could partially compensate for loss of STIM1-mediated SOCE in *STIM1* ^*PKO*^ PNs. This negative regulation of SOCE by SEPT7 in PNs could further modulate synaptic wiring and cerebellar circuit function in *STIM1* ^*PKO*^ mice. Our findings are relevant in the context of deciphering the therapeutic potential of SEPT7 inhibitors for neurodegenerative conditions where Ca^2+^ dyshomeostasis is observed over time.

## Data Availability

The original contributions presented in the study are included in the article/Supplementary Material, and further inquiries can be directed to the corresponding author.

## Supplementary Material

Supplementary Figure S1 Analysis of gene expression in *SEPT7*–*STIM1* knockout Purkinje neurons (related to Figure 3) **(A)** Bar graph showing relative fold changes in expression levels of the indicated genes of various control genotypes of *STIM1* ^*PKO*^; *SEPT7* ^*PHet*^. Red asterisks above each bar represent statistically significant groups compared between *STIM1* ^*PKO*^ (pink bars) and *STIM1* ^*flox/flox*^ *; SEPT* ^*flox/+*^(light blue bars). **(B)** Bar graph showing relative gene expression changes of the indicated genes of various control genotypes of *STIM1* ^*PKO*^ *; SEPT7* ^*PKO*^. Red asterisks over each bar represent statistically significant groups compared between *STIM1* ^*PKO*^(pink bars) and *STIM1* ^*flox/flox*^; *SEPT* ^*flox/flox*^(blue bars). Fold changes were normalized to *GAPDH*. Data are presented as mean ± SEM, **p* < 0.05, ***p* < 0.01; One-way ANOVA with post hoc Tukey’s test. All measurements are taken by qRT-PCR of cDNA prepared from RNA isolated from microdissected Purkinje layers (*n* = 3 mice for all groups and *n* = 4 for *STIM1* ^*PKO*^; *SEPT7* ^*PHet*^, age: 1-year-old mice). *Itpr1*, inositol 1,4,5-trisphosphate receptor 1*, Orai3*—Orai3; *Casq2*, calsequestrin 2, *S100b*—S100b; *Cacng5*, calcium voltage-gated channel auxiliary subunit gamma 5; *Kctd17*, pPotassium channel tetramerization domain containing 17; *Vamp1*, vesicle-associated membrane protein 1; *Syt11*, synaptotagmin 11, *Setd6*, SET domaincontaining 6, *Gapdh*, glyceraldehyde 3-phosphate dehydrogenase.

Supplementary Figure S2 Experimental setup of rotarod assay with mice of various genotypes of *SEPT7*–*STIM1* mutant strain (Related to Figure 5) Snapshots of mice of indicated genotypes on an accelerated rotarod at 0, 45, and 102 s. Lane 1–*STIM1* ^*flox/flox*^ *; SEPT7* ^*flox/flox*^ (wild), Lane 2–*STIM1* ^*PKO*^, Lane 3–*STIM1* ^*PKO*^, Lane 4–*STIM1* ^*PKO*^; *SEPT7* ^*PHet*^, Lane 5–*STIM1* ^*PKO*^ *; SEPT7* ^*PKO*^. All animals were aged 6 months. Mice that have fallen from the accelerating rod are indicated by red arrows.

Supplementary Video S1 Rotarod assay of *SEPT7*–*STIM1* mutant mice strain (related to Figure 5). Video of the rotarod assay with mice of indicated genotypes. Lane 1: *STIM1* ^*flox/flox*^; *SEPT7* ^*flox/flox*^ (wild), Lane 2: *STIM1* ^*PKO*^, Lane 3: *STIM1* ^*PKO*^, Lane 4: *STIM1* ^*PKO*^; *SEPT7* ^*PHet*^, Lane 5: *STIM1* ^*PKO*^; *SEPT7* ^*PKO*^. All mice were 6 months old in this video.
