# Supplementary figures and images for: Deficits Associated With Loss of STIM1 in Purkinje Neurons Including Motor Coordination Can Be Rescued by Loss of Septin 7
Source: Front Cell Dev Biol. 2021 Dec 21;9:794807. doi: 10.3389/fcell.2021.794807 (PMC8724567; doi:10.3389/fcell.2021.794807)

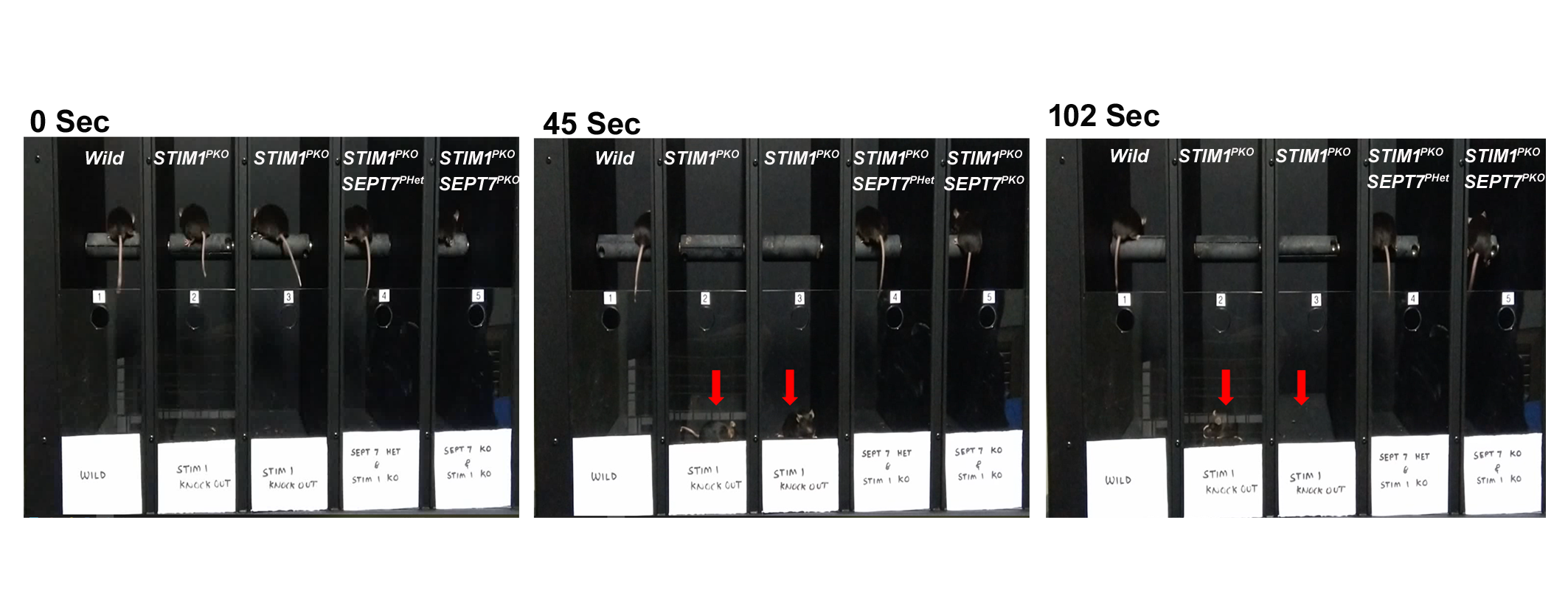

Supplement: Supplementary file 1 [file Image2.TIF]

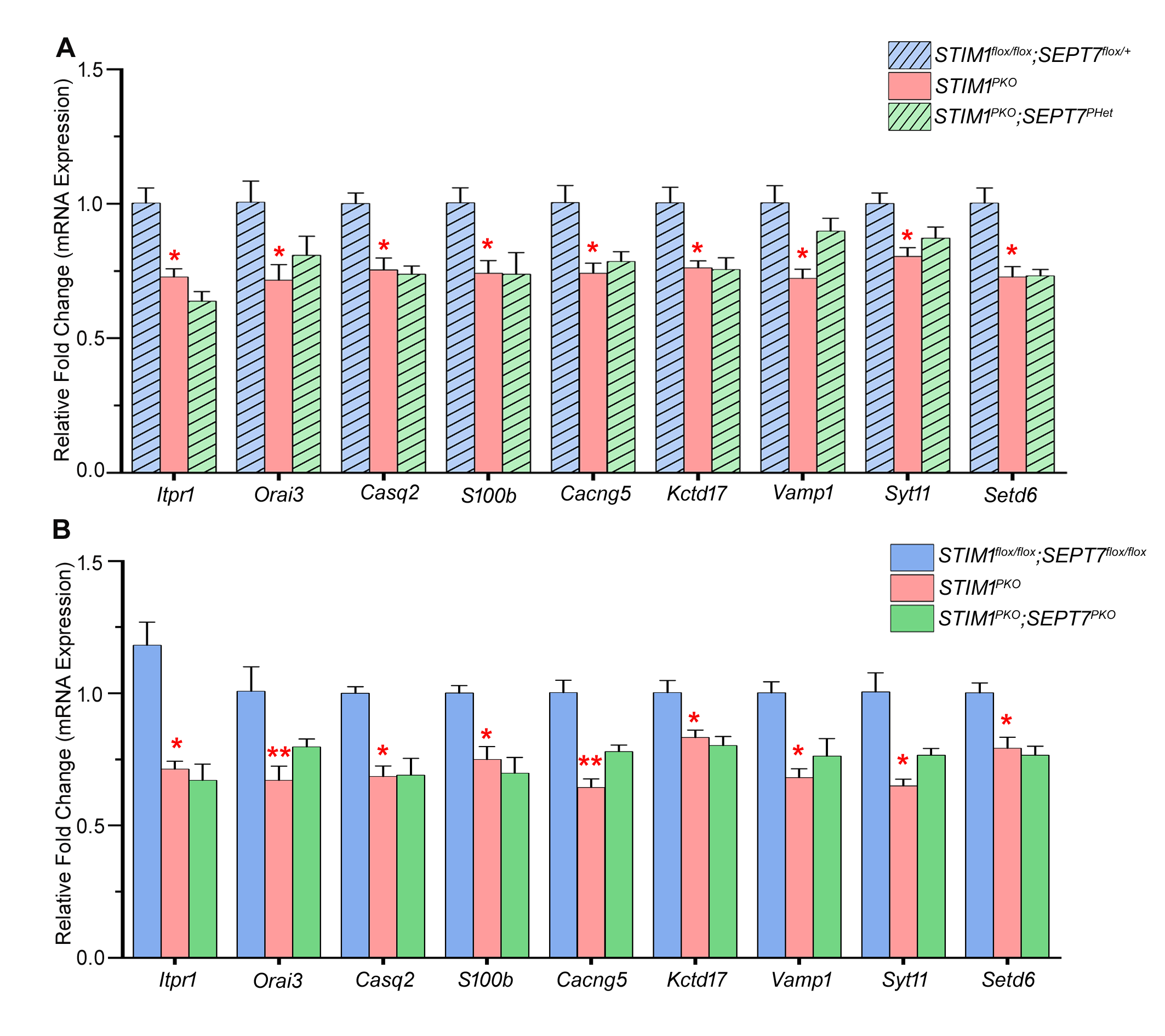

Supplement: Supplementary file 2 [file Image1.TIF]
